# Electrolyte abnormalities and clinical outcomes in children aged one month to 13 years hospitalized with acute gastroenteritis in two large referral hospitals in Botswana

**DOI:** 10.1371/journal.pgph.0004588

**Published:** 2025-05-08

**Authors:** Anita A. Kinasha, Jeffrey M. Pernica, Francis M. Banda, David M. Goldfarb, Henry D. Welch, Andrew P. Steenhoff, Sarah A. MacLean

**Affiliations:** 1 Aga Khan Hospital, Dar es Salaam, Tanzania; 2 Department of Paediatric and Adolescent Health, Faculty of Medicine, University of Botswana, Gaborone, Botswana; 3 Department of Paediatrics, McMaster University, Hamilton, Ontario, Canada; 4 Section of Infectious Diseases, Nationwide Children’s Hospital, Columbus, Ohio, United States of America; 5 Department of Pathology and Laboratory Medicine, University of British Columbia, Vancouver, Canada; 6 Department of Paediatrics, Baylor College of Medicine, Houston, Texas, United States of America; 7 Global Health Center, Children’s Hospital of Philadelphia, Philadelphia, Pennsylvania, United States of America; 8 Division of Infectious Diseases, Children’s Hospital of Philadelphia, Philadelphia, Pennsylvania, United States of America; 9 Department of Pediatrics, Perelman School of Medicine, University of Pennsylvania, Philadelphia, Pennsylvania, United States of America; Indian Institute of Public Health Shillong, INDIA

## Abstract

Acute gastroenteritis (AGE) is a common childhood disease, with a median of 2.5 diarrhoea episodes per child per year in both low- and middle-income countries. Morbidity and mortality from AGE result from a number of causes, including electrolyte abnormalities. This study characterized children hospitalized for AGE in Botswana with and without electrolyte abnormalities. This was a prospective observational study of children under the age of 13 years who were admitted with AGE in Princess Marina Hospital (PMH) and Nyangabgwe Referral Hospital (NRH) between May 2011 and April 2013. All children with serum electrolyte values obtained within 48 hours of admission were included. Patient characteristics and prevalence of electrolyte abnormalities were described. Risk factors for mortality were explored using univariate and multivariate logistic regression analyses. Among 544 patients, 37% had electrolyte abnormalities, namely hyponatraemia (9%), hypernatraemia (12%) and hypokalaemia (16%). Patients with hypernatraemia were younger (median age 6 months) compared to those with normal electrolytes (median age 9 months, p < 0.001). Patients with hypokalaemia presented after a longer duration of diarrhoea (median 4 days) compared to those with normal electrolytes (median 2 days, p < 0.001). Length of stay was longer in hypokalaemic (5 days) and hyponatraemic (5 days) patients compared to patients with hypernatraemia (3 days) and those without electrolyte abnormalities (3 days, p < 0.002). Those with malnutrition were significantly more likely to have electrolyte imbalances, with 19% having hypokalaemia. In multivariate analysis, the strongest predictors of mortality were malnutrition (OR 4.3; 95% CI 1.44-12.9); hypokalaemia (OR 5.5; 95% CI 1.84-16.6) and hypernatraemia (OR 3.9; 95% CI 1.11-13.5). Given the global impact of paediatric AGE, it is important that clinicians take additional care and precautions when admitting children with AGE and hypokalaemia, hypernatraemia, or malnutrition, as these increase the length of stay and odds of mortality.

## Introduction

Globally, acute gastroenteritis (AGE) is the second leading cause of death in young children after the neonatal period [1]. Gastroenteritis is defined as inflammation of the mucosal lining of the gastrointestinal tract which leads to vomiting and/or diarrhoea [2,3]. Diarrhoea is defined by the World Health Organization (WHO) as passage of three or more loose or watery stools per day or more frequent passage of stool than normal [4,5]. AGE is differentiated from persistent gastroenteritis by it lasting less than 14 days [4,5]. Severe AGE is that which causes hospital admission [4]. In 2016, about 90% of diarrhoeal deaths among children younger than 5 years occurred in South Asia and Sub-Saharan Africa [3]. Diarrhoeal illnesses are also known to cause long-term morbidity, leading to conditions including undernutrition and cognitive impairment [6]. Children under the age of five years are particularly at risk, with studies reporting a decreasing prevalence of diarrhoeal disease in children with increasing age [7–9].

In general, low and middle-income countries (LMIC) have higher rates of hospital admissions, morbidity and mortality from gastroenteritis compared to high-income countries (HIC) [7]. In Africa, among children under 5 years of age, there were between 2.04 and 2.85 episodes of diarrhoea per child per year in 2016 [10]. Factors contributing to higher rates of AGE in LMIC include undernutrition, increased vulnerability to infections, poor education, lower socio-economic status, the growing trend of breastmilk substitutes, poor health services, poor sanitation and unavailability of clean water [11,12]. In 2018 in Botswana, diarrhoea was the second leading cause of under-five mortality excluding neonates, accounting for 12% of deaths [13]. Between 2011 and 2013 at Princess Marina Hospital (PMH) and Nyangabgwe Referral Hospital (NRH), Botswana’s two largest hospitals, children with severe AGE had an in-hospital mortality rate of 4% [14]. These data confirm that AGE is a major cause of under-five morbidity and mortality in LMIC, including Botswana.

It is widely known that electrolyte abnormalities frequently occur in children with severe AGE with estimates varying between 30% to 60% [15–20]. Early mortality in patients with AGE usually occurs due to dehydration, which results from loss of fluid and electrolytes in diarrhoeal stools [21,22]. A study of AGE in Iran found 41.6% of participants had sodium abnormalities and 13% had hypokalaemia. In that study, the concentration of oral rehydration solution (ORS) given prior to admission played an important role in the type and severity of electrolyte abnormalities [20]. Both morbidity and mortality may be exacerbated by co-existent electrolyte abnormalities [19]. In Botswana, a number of studies have described the epidemiology and aetiology of AGE in children [14,23,24], but there have been no Botswana-based studies which explored the profile of serum electrolytes in children with AGE, the severity of electrolyte abnormalities seen in association with AGE, risk factors associated with severe electrolyte abnormalities, and clinical outcomes. Moreover, there is a paucity of data from the African continent on this topic. The primary objective of this study was to determine the rates of electrolyte abnormalities in a cohort of children hospitalized with AGE at two large referral hospitals. The secondary objective was to identify covariates associated with electrolyte abnormalities and clinical outcomes among children with and without electrolyte abnormalities.

## Methods

### Study setting and design

This was a prospective observational study conducted using an existing set of deidentified data collected between May 2011 and April 2013. The larger study enrolled 671 children admitted for severe acute gastroenteritis and analyzed stool samples to detect common enteropathogens. All participants were followed through hospital discharge and data on clinical outcomes was recorded. Inclusion criteria included being less than 13 years of age and having acute diarrhoea (defined as at least 3 loose stools or a single loose stool with at least 2 episodes of emesis in any 24-hour period prior to enrolment) for less than 14 days. Participants were admitted to the two largest public referral hospitals in Botswana, Princess Marina Hospital (PMH) and Nyangabgwe Referral Hospital (NRH) [14]. PMH has an average of about 130 admissions per month with 10% of these being children with AGE. Both hospitals follow protocols for community-acquired AGE based on WHO guidelines. These protocols include fluid resuscitation, zinc supplementation, and targeted blood investigations including full blood counts, electrolytes, and renal function tests within 48 hours of admission. At both hospitals, the decision to test electrolytes was ultimately at the discretion of the admitting clinician.

#### Inclusion criteria.

Children were included if they were less than 13 years old, admitted with less than 14 days of diarrhoea (defined as at least 3 loose stools or a single loose stool with at least 2 episodes of emesis in any 24-hour period prior to enrolment), and had electrolytes measured upon admission.

#### Exclusion criteria.

Patients were excluded if they did not have electrolyte values recorded on admission. They were also excluded if they had nosocomial infections, defined as developing diarrhoea more than 48 hours after admission or being readmitted with diarrhoea within 7 days of discharge from a previous hospital admission.

### Data analysis

Covariates used for the analysis included age, gender, method of feeding, HIV status, presence of vomiting, duration of vomiting, severity of vomiting, duration of diarrhoea, severity of diarrhoea, use of antibiotics at admission, presence and type of malnutrition, traditional medicine use, and serum sodium, potassium, urea and creatinine. For the multivariate analysis, electrolyte abnormalities were classified in order to create three distinct groups: hyponatraemia, defined as serum sodium <135 mmol/L, with normal potassium; hypernatraemia, defined as serum sodium >145 mmol/L, with normal potassium; and hypokalaemia, defined as serum potassium <3.5 mmol/L regardless of sodium level. Hyperkalaemia was not included in the multivariate analysis because in clinical practice it is not a common abnormality associated with AGE, but rather a result of the test being performed on a haemolysed blood sample, hence providing a falsely elevated potassium level. To determine whether there were differences in demographic characteristics, clinical history, and outcomes between children with different electrolyte abnormalities, either chi-square tests or Fisher’s exact test were used for categorical independent variables; t-tests or Kruskal-Wallis equality-of-populations testing were used to determine whether continuous variables (i.e., duration of diarrhoea, length of stay) were associated with categorical variables of interest.

The final analysis sought to determine which covariates were associated with death in the cohort. Certain covariates of interest (age, sex, HIV status, vomiting, diarrhoea, malnutrition, and electrolyte abnormalities) were run as univariate logistic regression and those predictive factors that were found to be associated with a p-value <0.2 were then entered into a forward stepwise multivariate logistic regression analysis. Only those covariates that led to a statistically significant (p < 0.05) improvement in the -2loglikelihood of the model were retained in the final multivariate model. The database was accessed on 19/03/2019 for this study and he data were analyzed using STATA version 11.0 (College Station, TX).

### Ethical considerations

Ethical approval was obtained from the ethics committees of the Botswana Ministry of Health, PMH, and the University of Botswana; written informed consent was obtained from the parent/guardian of each participant below 18 years of age.

## Results

Among the 671 children enrolled in the database, 544 (81%) had electrolyte values recorded on admission and were included in the study. Their demographic data, clinical characteristics and electrolyte abnormalities are summarized in Table 1. The median age was 8.6 months (IQR [interquartile range] 5–14 months) and the majority of patients were less than 12 months of age. Two thirds of patients were admitted to PMH and 56% were males. Almost half of patients (47%) were HIV negative/unexposed, one quarter (26%) were exposed but uninfected, and 5% were HIV positive. Among those <6 months, most patients (72%) were exclusively formula fed. In terms of clinical presentation, 18% of children had vomiting in addition to diarrhoea and 9% had severe acute malnutrition (SAM) as per WHO standards.

**Table 1:** Demographic characteristics, clinical presentation, and electrolyte abnormalities among children with severe acute gastroenteritis (N = 544).

Variable	n	Category	Frequency (%)	Median (IQR)
***Demographics***				
Age at presentation (years)	524			8.6 (5.0 – 14.3)
		≤12mo	357 (68)	
		>12mo and ≤ 60mo	161 (31)	
		>60mo and ≤ 13yrs	6 (1)	
Hospital	542	Princess Marina Hospital	364 (67)	
		Nyangabgwe Referral Hospital	178 (33)	
Gender	541	Male	302 (56)	
HIV status	543	Negative, unexposed	253 (47)	
		Exposed, uninfected	141 (26)	
		Unknown (high risk)	61 (11)	
		Unknown (low risk)	59 (11)	
		Positive	29 (5)	
Method of feeding (children <6 months)	159	Exclusive formula	115 (72)	
		Exclusive breastfeeding	28 (18)	
		Mixed feeding	16 (10)	
Household access to water	498	Community tap	177 (36)	
		Home	321 (64)	
***Clinical presentation***				
Duration of diarrhoea (days)	501			3 (1 – 4)
Presence of vomiting	515		95 (18)	
Duration of vomiting (days)	413			2 (1 – 4)
Presence of SAM	357		33 (9)	
Use of antibiotics	521		332 (64)	
Use of traditional medicines	110		60 (55)	
***Electrolyte abnormalities***				
Serum sodium (normal 135 – 145 mmol/L)	524			138 (133.6 – 144)
		Low (<135)	60 (11)	
		Normal	381 (73)	
		High (>145)	83 (16)	
Serum potassium (normal 3.5 – 5.5 mmol/L)	524			4.3 (3.8 – 5)
		Low (<3.5)	89 (17)	
		Normal	368 (70)	
		High (>5.5)	67 (13)	
Serum urea (normal ≤8 mmol/L)	490			5.6 (3.3 – 9)
		Normal	333 (68)	
		High	157 (32)	
Serum creatinine (normal ≤70 μmol/L)	436			32 (23 – 51)
		Normal	362 (83)	
		High	74 (17)	
***Outcomes***				
Length of stay (days)	540			4 (2 – 6)
Death	537		28 (5)	

As shown in Table 2, 37% of patients had electrolyte abnormalities. Of these, 9% had hyponatraemia with a normal potassium level, 12% had hypernatraemia with a normal potassium level, and 16% were hypokalaemic. There were statistically significant differences in the age distributions of the participants in each of the electrolyte groups (p value <0.001). In particular, almost all (95%) of children with hypernatraemia were ≤12mo. There were no significant differences in gender, HIV status, or method of feeding of the patients with electrolyte abnormalities compared to those without. However, household water source was associated with electrolyte abnormalities (p = 0.003) with higher rates of hyponatraemia and hypokalaemia amongst those using a community tap.

**Table 2:** Characteristics and clinical outcomes of children with severe acute gastroenteritis with and without electrolyte abnormalities.

Variable	n	Category	Hypo-natraemia	Hyper-natraemia	Hypo-kalaemia	Normal electrolytes	p
Total	544		47 (9)	66 (12)	89 (16)	342 (63)	
Age at presentation	524	≤12mo	26 (58)	61 (95)	52 (62)	218 (66)	<0.001
		>12mo and ≤ 60mo	19 (42)	3 (5)	29 (35)	110 (33)	
		>60mo and ≤ 13yrs	0 (0)	0 (0)	3 (4)	3 (1)	
Gender	541	Male	26 (57)	36 (55)	51 (57)	189 (56)	0.990
		Female	20 (43)	30 (46)	38 (43)	151 (44)	
HIV status	543	Negative, unexposed	12 (26)	28 (42)	41 (46)	172 (50)	0.120
		Exposed, uninfected	18 (38)	21 (32)	21 (24)	81 (24)	
		Unknown (high risk)^1^	8 (17)	9 (14)	8 (9)	36 (11)	
		Unknown (low risk)^2^	6 (13)	6 (9)	10 (11)	37 (11)	
		Positive	3 (6)	2 (3)	9 (10)	15 (4)	
Method of feeding (children <6mo)	159	Exclusive formula	11 (69)	22 (68)	11 (69)	71 (75)	0.820
		Exclusive breastfeeding	4 (25)	5 (16)	4 (25)	15 (16)	
		Mixed feeding	1 (6)	5 (16)	1 (6)	9 (9)	
Household access to water	498	Community tap	20 (51)	20 (32)	39 (49)	98 (31)	0.003
		House tap	19 (49)	43 (68)	40 (51)	219 (69)	
Duration of diarrhoea^*^	501	–	3 (2 – 3)	2 (2 – 4)	4 (2 – 7)	2 (1 – 4)	<0.001
Presence of vomiting	515	Yes	29 (69)	53 (82)	73 (90)	265 (81)	0.040
		No	13 (31)	12 (18)	8 (10)	62 (19)	
Duration of vomiting^*^	413	–	2 (1 – 3.5)	2 (2 – 4)	3 (2 – 4)	2 (1 – 3)	0.002
Presence of SAM	357	Yes	3 (12)	2 (4)	11 (19)	17 (8)	0.030
		No	22 (88)	54 (96)	48 (81)	200 (92)	
Use of antibiotics	521	Yes	40 (89)	40 (63)	65 (77)	187 (57)	0.001
		No	5 (11)	23 (37)	19 (23)	142 (43)	
Use of traditional medicines	110	Yes	7 (41)	9 (75)	18 (58)	26 (52)	0.320
		No	10 (59)	3 (25)	13 (42)	24 (48)	
Length of stay^*^	540	–	5 (3 – 8)	3 (2 – 6)	5 (2 – 8)	3 (2 – 6)	0.002
Death	537	Yes	2 (4)	6 (9)	10 (12)	10 (3)	0.006
		No	43 (96)	58 (91)	74 (88)	327 (97)	

*Median (IQR) in days; ^1^HIV status unknown (low risk) defined as mother denied or unsure about exposure, child not tested; ^2^HIV status unknown (high risk) defined as mother positive for HIV and child not tested; SAM, severe acute malnutrition.

There were statistically significant differences in the duration of diarrhoea (p < 0.001) and vomiting (p = 0.002) observed between the groups. In both cases, the hypokalaemia group had the longest duration of symptoms. There were statistically significant differences in the proportion of children with severe acute malnutrition between the groups (p = 0.03); the hypokalaemia group had the largest proportion of malnourished children. There were also statistically significant differences in length of stay between groups (p = 0.002), with a median length of stay of 5 days in both the hypokalaemia (IQR 2–8) and hyponatraemia (IQR 3–8) groups. Finally, there were statistically significant differences in case-fatality rates between groups (p = 0.006), with the most mortality (12%) observed in the hypokalaemia group.

Table 3 compares patients with sodium abnormalities to those with normal sodium, showing statistically significant differences in the age at presentation between the groups (p = 0.001). Of note, almost all patients with hypernatraemia (95%) were under 12 months of age.

**Table 3:** Characteristics and clinical outcomes of children with severe acute gastroenteritis with and without sodium abnormalities.

Variable	n	Category	Hypo-natraemia	Hyper-natraemia	Normal sodium	p
Total	544		60 (11)	83 (15)	401 (74)	
Age at presentation	524	≤12mo	31 (54)	76 (95)	250 (65)	0.001
		>12mo and ≤ 60mo	25 (44)	4 (5)	132 (34)	
		>60mo and ≤ 13yrs	1 (2)	0 (0)	5 (1)	
Gender	541	Male	34 (58)	47 (57)	221 (55)	0.938
		Female	25 (42)	36 (43)	178 (45)	
HIV status	543	Negative, unexposed	17 (28)	35 (42)	201 (50)	0.060
		Exposed, uninfected	22 (37)	25 (30)	94 (24)	
		Unknown (high risk)^1^	8 (13)	13 (16)	40 (10)	
		Unknown (low risk)^2^	8 (13)	8 (10)	43 (11)	
		Positive	5 (8)	2 (2)	22 (5)	
Method of feeding (children <6mo)	159	Exclusive formula	13 (72)	25 (64)	77 (75)	0.588
		Exclusive breastfeeding	4 (22)	8 (21)	16 (16)	
		Mixed feeding	1 (6)	6 (15)	9 (9)	
Household access to water	498	Community tap	27 (55)	28 (35)	122 (33)	0.012
		House tap	22 (45)	52 (65)	247 (67)	
Duration of diarrhoea^*^	501	–	3 (2 – 3.5)	3 (2 – 5)	2 (1 – 4)	0.192
Presence of vomiting	515	Yes	38 (73)	68 (83)	314 (82)	0.268
		No	14 (27)	14 (17)	67 (18)	
Duration of vomiting^*^	413	–	2 (1 – 4)	3 (2 – 4)	2 (1 – 3)	0.020
Presence of SAM	357	Yes	4 (13)	2 (3)	27 (11)	0.073
		No	27 (87)	69 (97)	228 (89)	
Use of antibiotics	521	Yes	51 (88)	51 (65)	230 (60)	<0.001
		No	7 (12)	28 (35)	154 (40)	
Use of traditional medicines	110	Yes	9 (43)	13 (72)	37 (52)	0.175
		No	12 (57)	5 (28)	34 (48)	
Length of stay^*^	540	–	5 (2 – 8)	3 (2 – 7)	3 (2 – 6)	0.070
Death	537	Yes	4 (7)	8 (10)	16 (4)	0.082
		No	55 (93)	75 (90)	379 (96)	

*Median (IQR) in days; ^1^HIV status unknown (low risk) defined as mother denied or unsure about exposure, child not tested; ^2^HIV status unknown (high risk) defined as mother positive for HIV and child not tested; SAM, severe acute malnutrition.

Table 4 compares patients with potassium abnormalities to those with normal potassium. There were statistically significantly differences in the duration of diarrhoea between the groups (p < 0.001); for example, those with hypokalaemia had a median of 4 days of diarrhoea (IQR 2–7), whereas those with normal potassium only had a median of 2 days of diarrhoea (IQR 1–4). There were also statistically significantly differences in the length of hospital stay between the different groups (p = 0.007). Patients with hypokalaemia had a median duration of stay of 5 days (IQR 2–8), whereas those with normal potassium had a median duration of stay of only 3 days (IQR 2–6).

**Table 4:** Characteristics and clinical outcomes of children with severe acute gastroenteritis with and without potassium abnormalities.

Variable	n	Category	Hypo-kalaemia	Hyper-kalaemia	Normal potassium	p
Total	544		89 (16)	67 (12)	388 (71)	
Age at presentation	524	≤12mo	52 (62)	54 (81)	251 (67)	0.036
		>12mo and ≤ 60mo	29 (35)	13 (19)	119 (32)	
		>60mo and ≤ 13yrs	3 (4)	0 (0)	3 (1)	
Gender	541	Male	51 (57)	40 (61)	211 (55)	0.646
		Female	38 (43)	26 (39)	175 (45)	
HIV status	543	Negative, unexposed	41 (46)	30 (45)	182 (47)	0.298
		Exposed, uninfected	21 (24)	18 (27)	102 (26)	
		Unknown (high risk)^1^	8 (9)	12 (18)	41 (11)	
		Unknown (low risk)^2^	10 (11)	6 (9)	43 (11)	
		Positive	9 (10)	1 (1)	19 (5)	
Method of feeding (children <6mo)	159	Exclusive formula	11 (69)	22 (71)	82 (73)	0.895
		Exclusive breastfeeding	4 (25)	6 (19)	18 (16)	
		Mixed feeding	1 (6)	3 (10)	12 (11)	
Household access to water	498	Community tap	39 (49)	23 (40)	115 (32)	0.011
		House tap	40 (51)	35 (60)	246 (68)	
Duration of diarrhoea^*^	501	–	4 (2 – 7)	2 (1.5 – 3)	2 (1 – 4)	<0.001
Presence of vomiting	515	Yes	73 (90)	48 (80)	299 (80)	0.086
		No	8 (10)	12 (20)	75 (20)	
Duration of vomiting^*^	413	–	3 (2 – 4)	2 (1 – 3)	2 (1 – 3)	<0.001
Presence of SAM	357	Yes	11 (19)	2 (4)	20 (8)	0.028
		No	48 (81)	47 (96)	229 (92)	
Use of antibiotics	521	Yes	65 (77)	39 (62)	228 (61)	0.015
		No	19 (23)	24 (38)	146 (39)	
Use of traditional medicines	110	Yes	18 (58)	8 (50)	34 (54)	0.862
		No	13 (42)	8 (50)	29 (46)	
Length of stay^*^	540	–	5 (2 – 8)	4 (2 – 8)	3 (2 – 6)	0.007
Death	537	Yes	10 (12)	4 (6)	14 (4)	0.015
		No	70 (88)	63 (94)	368 (96)	

*Median (IQR) in days; ^1^HIV status unknown (low risk) defined as mother denied or unsure about exposure, child not tested; ^2^HIV status unknown (high risk) defined as mother positive for HIV and child not tested; SAM, severe acute malnutrition.

In the multivariable logistic regression analysis (Table 5), malnutrition was associated with 4-fold higher odds of mortality (p = 0.009). Also, a statistically significant association between electrolyte abnormality and mortality was noted. Children with hypokalaemia had 5 times higher odds of mortality than those without electrolyte abnormalities (p = 0.002), whereas those with hypernatremia had almost 4 times higher odds of mortality than those without electrolyte abnormalities (p = 0.03).

**Table 5:** Univariate and multivariate logistic regression analysis of the factors associated with mortality in children with severe acute gastroenteritis.

Risk factor		Univariate		Multivariate	
		OR (95% CI)	p	OR (95% CI)	P
Age	≤12mo	*Reference*			
	>12mo and ≤ 60mo	0.52 (0.19 – 1.40)	0.19	–	–
	>60mo and ≤ 13yrs	3.16 (0.35 – 28.3)	0.30	–	–
Gender	Male	0.98 (0.45 – 2.13)	0.96	–	–
	Female	*Reference*			
HIV status	Negative, unexposed	*Reference*			
	Exposed, uninfected	0.89 (0.33 – 2.42)	0.82		
	Unknown (high risk)^1^	2.24 (0.81 – 6.25)	0.12		
	Unknown (low risk)^2^	1.08 (0.30 – 3.96)	0.91	–	–
	Positive	0.71 (0.089 – 5.65)	0.75		
Presence of SAM	Yes	4.71 (1.68 – 13.2)	0.003	4.31 (1.44 – 12.9)	0.009
	No	*Reference*			
Use of traditional medicines	Yes	1.51 (0.42 – 5.51)	0.53	–	–
	No	*Reference*			
Presence of vomiting	Yes	0.41 (0.18 – 0.95)	0.04	–	–
	No	*Reference*			
Duration of vomiting	Each incremental day	1.10 (0.91 – 1.33)	0.31	–	–
Duration of diarrhoea	Each incremental day	1.14 (1.02 – 1.28)	0.02	–	–
Hospital site	PMH	1.49 (0.62 – 3.57)	0.38	–	–
	NRH	*Reference*			
Electrolyte abnormality	None	*Reference*			
	Hyponatraemia only	1.49 (0.32 – 7.01)	0.616	1.31 (0.15 – 11.7)	0.800
	Hypernatraemia only	3.27 (1.15 – 9.33)	0.03	3.87 (1.11 –13.5)	0.030
	Hypokalaemia	4.19 (1.69 – 10.4)	0.002	5.53 (1.84 – 16.6)	0.002

OR, odds ratio; CI, confidence interval; ^1^HIV status unknown (low risk) defined as mother denied or unsure about exposure, child not tested; ^2^HIV status unknown (high risk) defined as mother positive for HIV and child not tested; SAM, severe acute malnutrition.

Note: Variables were included in the multivariate model if p < 0.2 on univariate analysis; those that did not lead to a statistically significant change in -2loglikelihood were removed

Hospital site was included in the analysis to account for one site (PMH) having a particular protocol for managing patients with AGE, which was not utilized at the other site (NRH). There was no statistically significant difference between the two sites (p = 0.38).

## Discussion

To the best of our knowledge, this is the first study on the prevalence of electrolyte abnormalities in children admitted with AGE in Botswana and one of the first in Sub-Saharan Africa [16]. The study describes associations of these electrolyte abnormalities to patient characteristics and clinical outcomes. The overall prevalence of electrolyte abnormalities was 37%. In the multivariate model, three factors were associated with higher odds of death: hypokalaemia (OR 5.5), hypernatraemia (OR 3.9) and malnutrition (OR 4.3).

### Prevalence of electrolyte abnormalities

In this study, 37% of subjects had electrolyte abnormalities. Hypokalaemia was the most common (16%) followed by hypernatraemia (12%) and hyponatraemia (9%). Hypokalaemia may be explained by the potassium loss that commonly occurs in children with diarrhoea and a subsequent physiological response to excessive fluid loss [25]. Hyponatraemia is commonly caused by fluid losses, which can lead to the release of antidiuretic hormone and subsequent retention of excessive water [25,26] whereas hypernatraemia may be caused by excessive fluid loss and ineffective rehydration [27]. The prevalence of hypokalaemia in malnourished children with AGE has been found in other studies in Pakistan and the Netherlands to be about 60%, with the rationale that these children tend to have an underlying subclinical hypokalaemia thought to be caused by a “whole body” chronic potassium depletion [28–30]. In contrast, studies of electrolyte abnormalities in children with AGE in Nigeria and India described hyponatremia as the most common abnormality in over 60% of children [16,19], whereas another study in Germany showed hypernatremia (77%) to be the commonest among patients with AGE, specifically children with rotavirus diarrhoea [31]. The differences with other studies may be attributed to different rates of ORS use prior to presentation to the hospital [32] and possibly use of incorrectly prepared infant formula [19]. Comparing AGE studies across continents has significant limitations, though, as there are many differences in factors such as local microbiology and rates of environmental enteropathy.

### Covariates associated with electrolyte abnormalities

In this study, hypernatraemia was more prevalent in younger patients. These findings are similar to those of a study on children admitted for severe diarrhoea in Bangladesh, where the median age for patients with hypernatraemia was seven months [32]. Hypernatraemia has been associated with dehydration in children younger than one year of age and is believed to be caused by ineffective breastfeeding or rehydration by the caregiver [27]. Younger children are unable to respond to thirst by drinking fluids on their own and are therefore at a greater risk of developing hypernatraemic dehydration [25].

Hypokalaemia is another important complication of AGE. Generally, a longer duration of diarrhoea is associated with hypokalaemia as was seen in this study, likely due to greater excretion of potassium in stool than sodium [25]. In this study, hypokalaemia was the most common abnormality amongst the 9% of subjects with SAM. Several studies have shown an association between malnutrition and electrolyte imbalances in children with AGE [30,33]. This is likely due to children with malnutrition being chronically potassium depleted even before they develop AGE [29,30,33]. Similarly, two studies in Pakistan showed about 60% of patients with malnutrition had hypokalaemia [28,29]. There were higher rates of hypokalaemia amongst those using a community tap. Prior studies have found that using a community tap is a risk factor for developing diarrhoea, but further research on the association between hypokalaemia and use of a community tap is warranted [3,12,34,35].

### Clinical outcomes

Electrolyte abnormalities are known to increase morbidity and mortality in patients with AGE [36]. In this study, both electrolyte abnormalities and malnutrition were associated with higher odds of mortality. Similarly, a study of children hospitalized for diarrhoea in Turkey showed an overall death rate of 6.75%, with hypokalaemia as a significant risk factor for mortality in univariate analysis [37]. A study in Bangladesh described a higher case fatality rate of 12% in hypernatraemia compared to 5% in hyponatraemia [32]. Furthermore, a study in Kenya found greater odds of death in patients with malnutrition (OR 3.5) and hypokalaemia (OR 2.5); that study also found 4.6-fold odds of death in patients with hyponatraemia [33]. Finally, in Mozambique, malnutrition was found to be an independent risk factor for death in patients with AGE with a hazard ratio of 4.1 in the multivariate analysis [21]. In combination, these studies reinforce the importance of having access to clinical electrolyte testing for any hospital that is managing patients with severe AGE. This is also supported by WHO which, in its Essential Diagnostics List, recommends electrolyte testing via automated chemistry analyser to be made available at the district hospital level and higher [38]. Nevertheless, recent surveys conducted in LMIC hospitals reveal that chemistry analysers are often not available [39]. The current WHO AGE management guidelines do not advocate for the routine measurement of serum electrolytes as these values are often misinterpreted, however in light of the study findings it may be necessary to revisit these guidelines [40,41]. In clinical practice, oral rehydration therapy (ORT) is generally recommended as the first-line treatment for paediatric gastroenteritis and dehydration, except in cases of severe dehydration where the use of intravenous fluids may be warranted. With the ability to measure serum electrolytes, clinicians would be better able to identify those patients with severe dehydration and provide the most appropriate options for their care. This is particularly important in LMIC where intravenous fluids are particularly limited [2,39].

### Study limitations

This study has some limitations. Although a prospective observational study with real-time data collection, there are unmeasured confounders including the possible impact of ORT, method of formula preparation, different aetiologies of diarrhoea as well as physical exam findings at presentation. Measurements of whether patients received ORT preadmission were not included, which could have affected their serum electrolyte profile at presentation. Similarly, 72% of patients under six months were exclusively formula fed, but the method of preparation was not recorded, which may have affected electrolyte values on admission.

## Conclusion

This study of electrolyte abnormalities in children admitted with AGE in Botswana found that hypokalaemia, hypernatraemia, and malnutrition were each associated with increased odds of mortality. It is imperative that clinicians take additional care and precautions when admitting children with the above risk factors. Given the global impact of paediatric AGE, further studies like this from other countries are needed to explore optimal management, minimize mortality, and obtain a clearer assessment of the impact of confounders that were unmeasured in this study.

## Supporting information

S1 Appendix Definitions of HIV status and severe acute malnutrition. (DOCX)

S2 Appendix AGE database. (XLS)
