# Supplementary material for: Electrolyte abnormalities and clinical outcomes in children aged one month to 13 years hospitalized with acute gastroenteritis in two large referral hospitals in Botswana
Source: PLOS Glob Public Health. 2025 May 8;5(5):e0004588. doi: 10.1371/journal.pgph.0004588 (PMC12061178; doi:10.1371/journal.pgph.0004588)
Supplement: S1 Appendix — (DOCX) [file pgph.0004588.s001.docx]

**Appendix**

HIV status:

- Unexposed: mother negative for HIV, child uninfected
- HIV exposed: mother positive for HIV, child uninfected
- HIV positive:
  - Child older than 18 months: positive rapid or positive HIV DNC PCR
  - Child younger than 18 months: positive HIV DNA PCR
- HIV unknown, low risk: mother denied or unsure about exposure, child not tested
- HIV unknown, high risk: mother positive for HIV and child not tested

Severe acute malnutrition (SAM):

- weight for height below -3 z-score of the median WHO growth standards
- presence of severe wasting
- presence of nutritional oedema
